# Viscosity Analysis of Battery Electrode Slurry

**DOI:** 10.3390/polym13224033

**Published:** 2021-11-21

**Authors:** Alex Cushing, Tianyue Zheng, Kenneth Higa, Gao Liu

**Affiliations:** 1 Energy Storage and Distributed Resources Division, Lawrence Berkeley National Laboratory, Berkeley, CA 94720, USA; alcushin@calpoly.edu (A.C.); tyzheng@lbl.gov (T.Z.); khiga@lbl.gov (K.H.); 2 Materials Engineering, California Polytechnic State University, San Luis Obispo, CA 93410, USA

**Keywords:** polymer composite, slurry, viscosity, coating, energy storage, lithium-ion rechargeable battery, composite electrode

## Abstract

We report the effects of component ratios and mixing time on electrode slurry viscosity. Three component quantities were varied: active material (graphite), conductive material (carbon black), and polymer binder (carboxymethyl cellulose, CMC). The slurries demonstrated shear-thinning behavior, and suspension properties stabilized after a relatively short mixing duration. However, micrographs of the slurries suggested their internal structures did not stabilize after the same mixing time. Increasing the content of polymer binder CMC caused the greatest viscosity increase compared to that of carbon black and graphite.

## 1. Introduction

Lithium-ion batteries are state-of-the-art rechargeable batteries that are used in a variety of demanding energy storage applications. Compared to other rechargeable batteries, lithium batteries are lightweight, have long cycle lives, and have high energy-to-weight ratios [1]. Electrode slurries are dispersions that are typically composed of conductive additives, polymer binders, and electrochemically active material particles that serve as reservoirs for lithium. They are coated onto conductive substrates and dried to form porous electrodes. Electrode pairs are assembled into lithium-ion batteries containing an electrolyte solution that allows the transport of lithium ions between electrodes. The two electrodes in a lithium-ion battery are of different compositions and provide energetically different environments for lithium. Lithium ions can travel between the two electrodes through the solution, while electrons instead travel through an external circuit as an electrical current.

Several factors influence the electrode fabrication process; we have chosen to investigate slurry viscosity. This is a key property affecting the consistency of the electrode performance. If slurry viscosity is too high, it can be difficult to produce uniform coatings, rendering the battery cycling time less predictable [2]. A high viscosity can also allow materials to clump together. This can cause an uneven reaction distribution on the electrode surface leading to hot spots during battery operation [2]. Low slurry viscosity, meanwhile, can cause runniness and pooling [3].

The predominant factor affecting slurry viscosity is composition. There are three key components to an electrode slurry: active material, conductive additive, and polymer binder [3,4]. The active material serves as a reservoir for lithium [5], while the conductive additive enhances electrical conductivity along the active material surfaces [6]. Graphite and acetylene carbon black are commonly used for active material and conductive additives, respectively. The polymer binder works to hold components together. Carboxymethyl cellulose (CMC) is a common polymer binder because it is water-soluble, has a high temperature resistance, and is electrochemically stable [7,8]. Styrene-butadiene rubber (SBR) has also been used as a binder. However, its contribution to viscosity is negligible at the commonly used weight percent [9]. Overall, the compositions of the electrode affect its conductivity, energy capacity, and stability [10].

Previous publications found that increasing solid content (active material, conductive additive, or polymer) will increase viscosity [11]. Studies have also found that CMC can play a large role in determining slurry viscosity [12]. However, there has been no research in comparing how the amount of conductive additive or active material affects viscosity. Furthermore, the effect of processing duration on rheology has not been explored either. While previous studies have used polyvinyl difluoride (PVDF) as the polymer binder, this study will be the first to examine the potential impact of CMC on battery slurry viscosity. It should be noted that the exact values of viscosity found will differ from those in industry. This is because viscosity is largely dependent on processing and equipment. The goal of this paper is to observe and quantify the tends of the individual slurry components’ effects on overall viscosity.

This paper will look specifically into how different preparation methods and components can influence slurry viscosity. Additionally, it will provide unique microscope images of graphite, carbon black, and CMC slurries. There will be four key factors in the electrode slurry fabrication process that will be analyzed: (1) how slurry viscosity varies with viscometer spindle speed; (2) how mixing duration affects slurry viscosity; (3) how the internal arrangement of slurries changes with mixing time; (4) how composition ratios affect slurry viscosity.

## 2. Materials and Methods

The active material used in this experiment was Imerys SFG 6 Mag-E graphite powder (Hitachi, Ltd., Chiyota, Japan) with a particle size of 6 μm diameter. The acetylene carbon black powder had a mean particle size of 50 nm (Denka, Tokyo, Japan). The polymer binder used in this experiment was carboxymethyl cellulose sodium salt (CMC) (Sigma-Aldrich, St. Louis, MO, USA), with weight average molecular mass of 250,000 Dalton and 0.7 degrees of substitution. Previous publications found that combining active material and conductive additive first, then polymer and solvent later in the mixing process can improve discharge capacity retention [13,14]. This will be the mixing order used for this experiment.

A 1% (percentages are expressed by weight) carboxymethyl cellulose (CMC) slurry was prepared as follows: 0.2 mg of dry CMC powder were added to 20 mL of water in a small jar. Following a similar process, 0.1 mg of carbon black powder was added to another 1% CMC slurry to make up a 1% CMC, 0.5% carbon black slurry. A third slurry was prepared by adding 9 mg of Mag-E graphite powder. Three additional slurries with the same compositions as the first three except with 2% CMC (0.4 mg of CMC) were also prepared. In summary, the following slurry compositions were prepared: 1% CMC; 1% CMC and 0.5% carbon black; 1% CMC, 0.5% carbon black, and 45% graphite; 2% CMC; 2% CMC and 0.5% carbon black; 2% CMC, 0.5% carbon black, and 45% graphite. Thirty zirconium oxide mixing balls (with 2 mm diameter) were added to accelerate the mixing process.

The slurries were mixed using a Paul O. Abbe Laboratory Jar Rolling Machine that ran at approximately 300 RPM for approximately 3 days. From each slurry, a small sample (approximately 5 mL) was extracted and evaluated in a Brookfield DV-E Viscometer with an HB-3 spindle. Every experiment described here was conducted at a room temperature of approximately 23 °C. The sample jars were then placed into the rolling machine for approximately one additional day, after which the viscosity measurements were repeated to check for consistency. Each test was repeated 3 times.

To test the effect of spindle speed, 1% and 2% CMC slurries with and without carbon black and graphite were tested. The slurries had been mixed for 3 days. The viscosity was reported at the highest spindle speed at which a consistent reading was obtained. For the 1% CMC mixture, spindle speed ranged from 2 to 35 RPM. The 2% CMC mixture was tested between 0.25 and 2 RPM as the readings were inconsistent at higher speeds.

To examine the influence of mixing duration on viscosity, 1% CMC slurries were prepared as before, but the samples were extracted after mixing for 30 min, 45 min, 1 h, 2 h, 3 h, 5 h, 18 h, and 3 days. At each time interval, viscosity was measured at a spindle speed of 10 RPM, after which micrographs were obtained. It was found that 10 RPM was the highest spindle speed at which all the 1% solutions gave stable readings.

To prepare the micrographs, a 1 mL sample of the slurry was placed in the middle of the microscope slide using a pipette. A coverslip was then placed on top. The slide was placed under an optical microscope and images were taken at various magnifications.

To examine the influence of compositional variation, slurries were prepared as before with approximately 3 days of mixing and were tested using the viscometer operating at 2 RPM, the highest readable speed for all samples. Above 2 RPM, the most viscous slurries did not give consistent readings.

## 3. Results and Discussion

**Figure 1:**
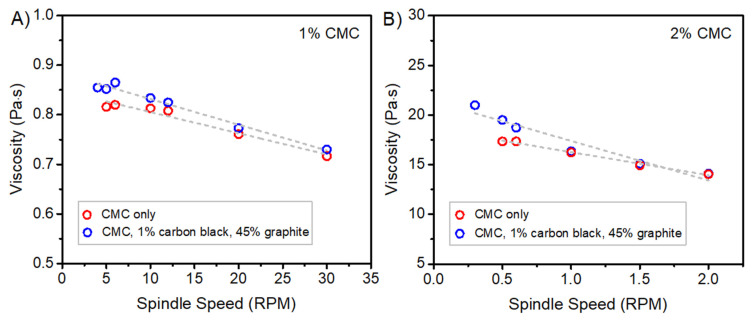
Viscosity versus spindle speed of (**A**) 1% carboxymethyl cellulose (CMC) and 1% CMC, carbon black and graphite slurries; (**B**) 2% CMC and 2% CMC, carbon black, and graphite slurries.

Figure 1 shows the viscosity of the slurries as a function of spindle speed. In each instance, the data were fit with a linear approximation. The consistency check between the 3-day sample and the 4-day sample did not show any directional bias. As the speed of the viscometer increased, the slurry viscosity tended to decrease, demonstrating shear-thinning behavior. Previous studies also found CMC electrode slurries to have similar properties [15,16]. In contrast, the viscosity of a Newtonian fluid does not depend on the shear rate. A possible explanation for the shear-thinning behavior is that as the shear rate of the viscometer increases, the arrangement of slurry components changes in a way that reduces mechanical shear resistance, thus lowering viscosity [16]. In addition, there could be a change in polymer chain alignment resulting in a disentanglement of the polymers in the slurry [15].

**Figure 2:**
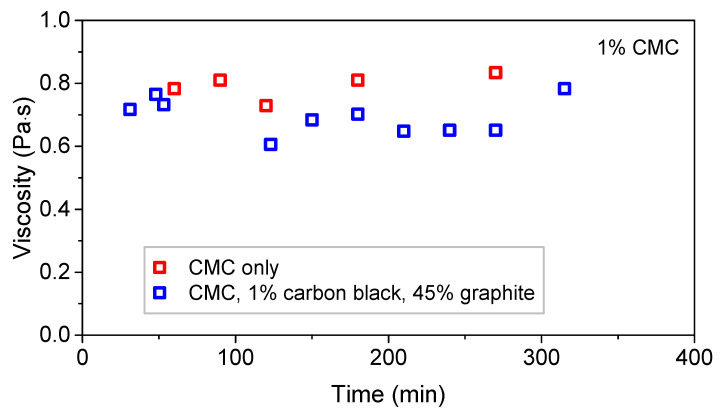
Viscosity versus mixing time of 1% CMC slurries at 10 RPM.

Figure 2 displays the effect of mixing time on the viscosity of a 1% CMC slurry and a 1% CMC, graphite, and carbon black slurry measured at a spindle speed of 10 RPM. 2% CMC slurries were also tested, but there was not a single spindle speed where all viscosity readings were stable. The viscosity at the 18 h and 3 day mixing mark were not shown as their values were within the ranges of the previously tested samples. Measured viscosity values appeared to be insensitive to mixing time. Thus, one might suspect the suspension properties have stabilized and no additional mixing is necessary. These results differed from those of the experiment conducted by Kim et al. [13], in which the researchers found a negative correlation in mixing duration and viscosity. This could be explained by the fact that their experiment involved different materials (PVDF as a polymer binder and LiCoO_2_ as active material) the viscometer spindle speed was 1000 RPM. The rate of dissolution of CMC powder in water depends on molecular weight [17], therefore, this study starts from a fully dissolved CMC in water solution. It is also possible that the interactions between the binder and active material of other binders (such as PVDF) were different than in the CMC slurries, requiring longer mixing times to stabilize.

**Figure 3:**
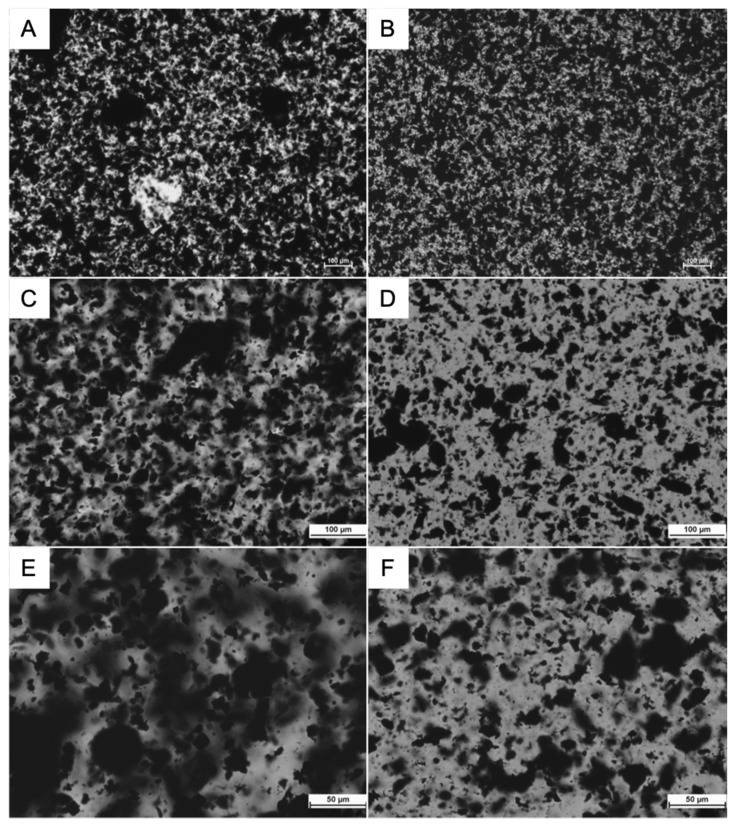
Microscope images of carbon black, 1% CMC, graphite after (**A**) 2 h of mixing (50×); (**B**) 4 h of mixing (50×); (**C**) 2 h of mixing (100×); (**D**) 4 h of mixing (100×); (**E**) 2 h of mixing (200×); (**F**) 4 h of mixing (200×).

These results can be further understood by examining optical microscope images of the slurry containing 1% CMC, carbon black, and graphite as shown in Figure 3. Images A, C, and E (left column) represent the slurry mixed for 2 h at magnifications of 50×, 100×, and 200×. Images B, D, and F (right column) represent the slurry that was mixed for 4 h at 50×, 100×, and 200× magnifications. All images were converted to grayscale. Dark regions are visible in all of the micrographs and presumably represent the solid components of the slurry: carbon black and graphite. Carbon black particle diameters range from 20 to 60 nanometers [18,19], so one would not expect to see individual particles in these micrographs. However, it is possible that they could be visible as clumps or in diffuse form, blocking light at sufficiently high local concentrations. Graphite particles, which are substantially bigger [20], could be inferred to make up most of the larger dense black spots throughout the images because the slurries are composed of 45% graphite compared to 0.5% carbon black by weight. Their densities are similar; carbon black has a density of 1.75 g/cm^3^ [21], whereas the density of graphite is 2.25 g/cm^3^ [22], so the graphite should occupy slightly more than 100 times the amount of volume occupied by carbon black when accounting for weight percent. However, carbon black could be much more evenly dispersed due to its smaller particle size.

Due to the difference in particle size, the sharply defined black clumps in all of the images are presumed to be graphite. We presume that the blurry, “cloudy” black regions in images C, E, and F are made up of the much smaller carbon black particles. In image A, the clear spot near the center of the image might be undissolved CMC.

Between images A and B (2 h and 4 h, 50×), the graphite arrangement in image B appeared more uniform. In image B, there were fewer large clumps and the solid material appeared to be more homogenous. This suggests that mixing was still incomplete at the 2 h mark. At a higher magnification, the cloudy regions visible in image C (2 h, 100×) were not visibly apparent in image D (4 h, 100×). The visible changes from E-F (200×) were in accordance with those from C-D. Once again, the cloudy regions were very apparent in image E (2 h, 200×), but were barely visible in image F (4 h, 200×). In both of these cases, the images taken after 4 h of mixing appeared to have more open space. One possibility is that the carbon black in the previously cloudy regions had been evenly dispersed into the open spaces. Another possibility is that the cloudy regions condensed into a denser form, such as surface coatings. Between images F and D, cloudy regions that were not easily seen in image D became evident at the higher magnification of image F. This suggests that as the slurries are mixed for longer periods, the cloudy carbon black regions continued to disperse, becoming less visible.

Drawing from these images and previous experiments, changes in internal arrangements seemed to not necessarily correspond with changes in viscosity measurements. The experiment in which mixing time was varied showed a viscosity stabilization after around 2 h of mixing, suggesting that no more mixing was necessary. However, while observing the micrograph images, given the notable differences between the 2 h and 4 h images, it seems that the slurry had not reached a final internal state after 2 h of mixing.

**Figure 4:**
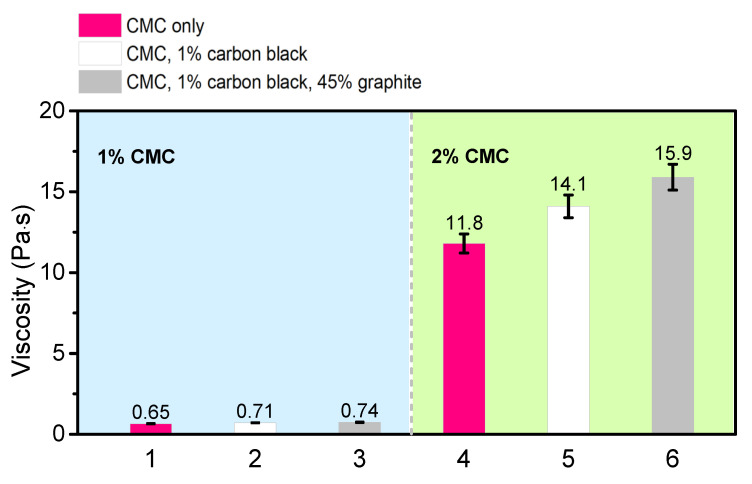
Viscosities of various slurry compositions.

The final experiment compared the effect of CMC, graphite, and carbon black on slurry viscosity (Figure 4). Slurries were tested at a spindle speed of 2 RPM and after 72 h of mixing. The numbers in the figure are averages of three trials, and error bars (ranging from 5 to 10%) show the standard deviations. Most notably, the addition of extra CMC caused a significant increase in viscosity in slurries with 2% CMC over slurries with 1% CMC. The addition of carbon black and graphite to the slurry had a relatively modest effect on viscosity, almost ten times less than that of the additional 1% CMC. The polymer binder clearly has the greatest impact on a per-weight basis. Although conventional PVDF and N-methyl-2-pyrrolidone (NMP)-based battery slurries might be significantly different from our CMC and water-based slurries, we note that Malvern Panalytical conducted a study using PVDF and NMP slurry and obtained a qualitatively similar conclusion. Their experiment found that a slurry composed of PVDF and NMP had a viscosity 200 times higher than a solution of just NMP [23]. We speculate the increased viscosity is due to the innate characteristics of the polymer. It acts as a glue and as the macromolecule entangles with itself and other slurry components, it creates a large resistant force against the viscometer [24].

## 4. Conclusions

This study examined the consequences of aspects of the battery electrode slurry preparation process on viscosity. Based on the experiments described here, it is evident that spindle speed, compositional ratios, and mixing time all influence slurry viscosity. The electrode slurries exhibited shear-thinning behavior, possibly due to agglomerate evolution and polymer chain disentanglement. Viscosity was largely unaffected by mixing time. While this might lead one to suspect that the slurry properties had stabilized within even the shortest mixing time tested (30 min), micrographs of a sample slurry composition suggested that internal component arrangement did not stabilize after even two hours of mixing. This suggests that stable slurry viscosity measurements are not sufficient to establish that the slurry microstructure has reached a steady state. Finally, CMC was confirmed to have a significantly larger impact on slurry viscosity than that of carbon black or graphite. Future experiments could examine the necessary mixing time to reach a steady state and analyze electrode porosity and electrochemical behavior.

## Data Availability

Original microscopy image available.
